# Sex-related differences in pain behaviors following three early life stress paradigms

**DOI:** 10.1186/s13293-016-0082-x

**Published:** 2016-06-10

**Authors:** Dawn K. Prusator, Beverley Greenwood-Van Meerveld

**Affiliations:** VA Medical Center, University of Oklahoma Health Science Center, Oklahoma, OK USA; Department of Physiology, University of Oklahoma Health Science Center, Oklahoma, OK USA; Oklahoma Center for Neuroscience, University of Oklahoma Health Science Center, BRC 272, 975 NE 10th St, Oklahoma, 73104 OK USA

**Keywords:** Early life stress, Visceral pain, Rats, Sex differences, Irritable bowel syndrome

## Abstract

**Background:**

Early life stress (ELS) serves as a risk factor for the development of functional pain disorders such as irritable bowel syndrome (IBS) in adults. Although rodent models have been developed to mimic different forms of ELS experience, the use of predominantly male animals across various rodent strains has led to a paucity of information regarding sex-related differences in the persistent effects of ELS on pain behaviors in adulthood. We hypothesized that the context or nature of ELS experience may interact with sex differences to influence the development of chronic pain.

**Methods:**

We employed three rodent models mimicking different facets of early life adversity to investigate the effects of ELS on pain perception in adulthood. To eliminate strain differences, all experiments were carried out using Long Evans rats. As neonates, male and female rat pups were exposed to maternal separation (MS), limited nesting (LN), or odor attachment learning (OAL). In adulthood, visceral sensitivity and somatic sensitivity were assessed at ~postnatal day 90 via quantification of visceromotor responses to colorectal distension and von Frey probing, respectively.

**Results:**

Following exposure to MS or LN, male rats developed visceral and somatic hypersensitivity compared to controls, whereas females subjected to the same paradigms were normosensitive. In the OAL model, females exposed to unpredictable ELS exhibited visceral but not somatic hypersensitivity. There were no observed differences in visceral or somatic sensitivity in male animals following OAL exposure.

**Conclusions:**

In summary, our data confirms that early adverse experiences in the form of MS, LN, and OAL contribute to the long-term development of heightened pain responsiveness in adulthood. Furthermore, this study indicates that sex-related vulnerability or resilience for the development of heightened pain perception is directly associated with the context or nature of the ELS experienced.

## Background

Irritable bowel syndrome (IBS) is a functional gastrointestinal (GI) disorder characterized by abdominal pain due to visceral hypersensitivity [1]. Moreover, visceral pain is often comorbid with abnormalities in somatic sensitivity, GI motility, and psychiatric disorders such as anxiety [2–4]. Currently, therapeutic options for the treatment of IBS are limited to treatment of symptoms due to our lack of knowledge concerning the etiology of functional pain. In an effort to understand how functional pain disorders may develop, clinical studies have been used to assess potential risk factors for the development of chronic pain and have indicated that stressful experiences in early life play a key role in the development of functional pain disorders such as IBS in adulthood [5, 6].

Patients with functional pain disorders are two to four times more likely to report a history of early life stress (ELS) during childhood [6]. ELS can be characterized by neglect, physical or psychological abuse, as well as an unhealthy attachment to an abusive caregiver. Each of these experiences can arise from a multitude of different circumstances such as socioeconomic standing, parental stress, and genetic predisposition [7–9]. Early life experiences are complex and highly variable, which makes the identification of mechanisms leading to the long-term consequences of ELS in humans a significant challenge. To better understand the etiology of adult pain pathologies, animal models have been developed to investigate the mechanisms by which ELS may lead to chronic visceral pain. Each rodent model of ELS was developed to recapitulate specific aspects of the human ELS experience to aid in the understanding of long-term health-related consequences. Validated models of ELS include (1) maternal separation (MS) developed to model neglect and abuse, (2) limited nesting (LN) developed to model abuse as a result of an impoverished environment, and (3) odor attachment learning (OAL) which models attachment to an abusive caregiver. These ELS models have been shown to induce heightened visceral pain in addition to changes in gut permeability, anxiety-like behaviors, and adult responses to stress [10–14]. However, the use of predominantly male animals in experimental models for female predominant conditions such as IBS limits the translational capacity of these studies. More specifically, although IBS affects between 3 and 32 % of the population [15], it is more commonly reported in females [16]. The lack of sex-related data from ELS paradigms restricts our ability to evaluate the breadth and usefulness of specific experimental protocols in studying mechanisms that underlie chronic pain. Additionally, the use of varying rodent strains within the same paradigm has limited our ability to clearly define the effects of each early life experience on adult pathologies observed across studies. Overall, inconsistent use of male and female animals and rodent strains limits our ability to fully compare the long-term effects of ELS on pain across studies. Therefore, the current study fills an important gap by performing a comprehensive and comparative analysis of several ELS models using male and female animals from a single rodent strain to investigate visceral and somatic pain thresholds in adulthood.

In the present study, we present parallel assessments of the MS, LN, and OAL models of ELS using male and female Long Evans rats to address that hypothesis that the development of chronic pain in adulthood is influenced by sex differences in either the vulnerability or resilience to a specific ELS experience. The findings from our study provide a platform for the future identification of sex-related differences in vulnerability and resilience to the long-term effects of ELS. Furthermore, our study aims to illustrate the translational relevance of each model to the clinical population.

## Methods

### Animals

Long Evans timed-pregnant rats were received from Charles River Laboratories ~embryonic day 9 on arrival and randomly assigned to treatment groups (*n* = 6/LN; *n* = 6/MS; *n* = 7/OAL) (Wilmington, MA). Housing conditions for temperature (23 °C) and light (12-h light/dark cycle from 7 AM to 7 PM) were controlled, and food (5053 Irradiated PicoLab Rodent Diet, LabDiet, St. Louis, MO) and water were available ad libitum. Ten to 12 days after arrival dams gave birth, which was designated as postnatal day (PND) 0. On PND1, litters were cross fostered within their respective groups (i.e., maternal separation with maternal separation controls) and culled to a maximum of 12 pups with equal male/female ratios when possible with 6 being the minimum litter size. All rats were housed under identical housing conditions to ensure the same sensory environment during growth into adulthood. The Oklahoma City VAMC Institutional Animal Care and Use Committee (IACUC) (1403-001) and the University of Oklahoma Health Sciences Center IACUC (14-156-I) approved all animal procedures. All animals were acclimated to the laboratory and experimental areas for 2 weeks prior to adult experiments.

### Neonatal LN model of ELS

Long Evans male and female pups were housed with dams on aspen bedding from PND0 through PND1, and the limited nesting protocol occurred PND2 through PND9 as described previously [17]. Pups and dams were housed under limited nesting conditions in cages with wire bottom flooring and a single paper towel for bedding material. During the limited nesting protocol, cages were not changed out and were checked three times daily to ensure all pups were still in the nest on top of the wire bottom. Pups and dams were returned to regular aspen bedding on PND10 and left undisturbed until weaning on PND22, when they were weighed and housed two per cage by sex and treatment until adulthood. Animals in the control group remained on regular bedding and were left undisturbed except during the weekly cage changes until weaning on PND22.

### MS model of ELS

Long Evans male and female pups were housed with dams on aspen bedding from PND0 through PND2. On PND2 through PND14, pups were separated from dams for 180 min per day between 09:00 AM and 12:00 PM [14]. Pups were removed and placed into an empty cage, which was then taken to an adjacent room and placed onto a heating blanket to maintain pup core temperature at 36–37 °C. At the completion of each 180-min separation period, pups were returned to home cage. Following the MS protocol, pups remained with dams until weaned at PND22, when they were weighed and housed two per cage by sex and treatment until adulthood. Animals in the control group remained on regular bedding and were left undisturbed except during the weekly cage changes until weaning on PND22.

### OAL model of ELS

As previously described [18], Long Evans male and female pups were housed with dams on aspen shaving bedding from PND0 through PND22. On PND8-12, pups were subjected to an odor-shock conditioning paradigm. Animals were conditioned six at a time divided into one of three treatment groups: (1) predictable odor-shock group, (2) unpredictable odor-shock group, and (3) odor-only control group. No more than two animals undergoing predictable OAL treatment were conditioned at a time to ensure the experimenter’s ability to immediately deliver the shock following odor presentation. Conditioning was performed using a series of 11 30-s peppermint odor presentations with 4-min inter-stimulus intervals. At the completion of the conditioning series, pups were returned to the nest and the process was repeated for the other half of the litter. Peppermint oil (Fisher Scientific, Nazareth, PA) was vaporized and odor presentations occurred at a 1:10 concentration of odor, administered at 2 L/min using a flow dilution olfactometer (Med Associates, Georgia, VT). Conditioning occurred during lights on 7 AM:7 PM. Durations of separation from mother were less than 1-h daily. Predictable odor-shock pups were subjected to a 0.5-mA shock (Coulbourn Instruments, Whitehall, PA) to the base of the tail during the final second of the odor presentation [19]. Unpredictable odor-shock pups received the same 0.5-mA shock to the base of the tail 2 min after the odor presentation. Odor-only controls were given a 30-s odor presentation only. During each conditioning series, a scale of 0 to 5 was used to score behavioral activation where 0 represented no movement and 5 represented the movement of all four limbs and the head. Behavioral activation was then quantified as the mean of the trials taken over the 5-day conditioning paradigm. Pre-odor and odor scores where then compared to assess the level of activation as an indication of a learned ability to predict the shock. A Y-maze was used to verify attachment learning following the conditioning protocol on PND13. Pups were given five trials, 1 min each, to make a choice between clean aspen bedding or the peppermint odor. Pups in the predictable group showed a preference for the peppermint odor (three or more of the five trials) (Table 1). Following the OAL protocol, pups remained with dams until weaned at PND22, when they were weighed and housed two per cage by sex and treatment until adulthood.

**Table 1:** Y-maze choices following neonatal conditioning

Learning verification: odor preference choices		
Treatment	Male	Female
Odor only	0.9 ± 0.3	1.0 ± 0.7
Predictable	3.3 ± 0.5**	3.7 ± 0.5**
Unpredictable	1.2 ± 0.8	1.2 ± 0.6

Values shown as mean ± SEM

**p < 0.01 vs odor-only control

Following neonatal conditioning, animals in the predictable ELS group exhibit a preference for the conditioned odor during Y-maze testing.

### Assessment of somatic sensitivity

In adult rats, somatic sensitivity was assessed on ~PND 90 via electronic von Frey test as previously described, following a 2-week acclimation to the lab and experimental areas [20–22]. All experiments occurred between 10 AM and 2 PM. After acclimating to the room for 30 min on the day of the experiment, rats were placed on an elevated mesh floor (12 mm × 12 mm grid), enclosed and unrestrained, in a clear plastic enclosure (21 × 27 × 15 cm). Once placed in the experimental apparatus, rats were allowed to acclimate for an additional 30 min prior to experimentation. An ElectroVonFrey filament (IITC Inc, Woodland Hills, CA) was applied to the plantar surface of the hindpaw from under the mesh floor. The filament was attached to a calibrated force transducer to digitally record the force required to cause withdrawal of the hind limb. The filament was applied until a brisk lifting of the paw, jumping, or sharp withdrawal of the hind limb was achieved. The average of three positive responses, each 5 min apart, was recorded as the value for each animal. A decrease in the threshold required for limb withdrawal was interpreted as increased somatic sensitivity.

### Assessment of visceral sensitivity

Colonic sensitivity was assessed in freely moving adult rats after maturation to adulthood ~PND 90 using clean standard rat cages with Sani-chip bedding. All experiments occurred between 10 AM and 2 PM. The visceromotor behavioral response (VMR) to colorectal distension (CRD) was measured by counting the number of abdominal contractions in response to isobaric distention at pressures of 0–60 mmHg [21–23]. An abdominal contraction consisted of longitudinal stretching of the body and visible contraction of the abdominal cavity. Following overnight fasting, rats were anesthetized by isoflurane inhalation (2–5 %) during the insertion of a 5-cm latex balloon catheter into the distal colon. With the catheters correctly in position, animals were allowed to recover and acclimate to the behavioral area for 30 min before colonic sensitivity was assessed. Following recovery, the catheter was connected to a Distender Series IIR barostat (G & J Electronics Inc.) for controlled, isobaric inflation of the colorectal balloon at 0, 20, 40, and 60 mmHg in randomized order. Each pressure was maintained for a period of 10 min during which time the number of abdominal muscle contractions were counted. A 10-min rest period was allowed between distensions.

### Estrus cycle characterization

To assess cycling in adult female rats, vaginal smears were collected three times weekly for 2 weeks before behavioral assessments began ~PND 76. Vaginal smears were also collected following each of the adult behavioral assessments. Vaginal cells were collected with cotton-tipped applicators moistened with purified water, inserted 1 cm into the vaginal opening and gently rotated to collect cells. Collected cells were then smeared onto microscope slides and the predominant cell types were identified using light microscopy. Estrus cycle phase was assigned (diestrus, metestrus, proestrus, or estrus) by identifying the predominant cell type and analyzed by grouping diestrus/metestrus and proestrus/estrus [24]. All females used for the current study were regularly cycling. Male animals were sham swabbed across the external perineum as a control for handling.

### Statistical analysis

Data are represented as the mean ± the standard error of the mean (SEM). A power calculation was used to determine necessary animal numbers. All data were tested for normality. The experimenter was not blinded to treatment. One-way analysis of variance (ANOVA) was used to compare odor preferences in the OAL model (*n* = 9–13/group) with a Bonferonni post hoc test. A two-way ANOVA was used to compare weaning weights and somatic sensitivity followed by a Bonferroni post hoc test to identify specific differences between sex and treatments (*n* = 9–15/group). VMR data was compared using separate two-way repeated measures ANOVAs (for each ELS, sex × pressure; for each sex, ELS × pressure) followed by a Bonferroni post hoc test (*n* = 9–15/group). The effects of estrus cycling were determined by comparing diestrus/metestrus vs proestrus/estrus within each separate group and then by comparing control vs ELS within diestrus/metestrus or control vs ELS in proestrus/estrus. The effect of estrus cycling on somatic sensitivity within each group was assessed using a Student’s *t* test to compare diestrus/metestrus vs proestrus/estrus. For the MS and LN data sets, a Student’s *t* test was used to compare control vs ELS in diestrus/metestrus or control vs ELS in proestrus/estrus. A one-way ANOVA was used to compare control vs ELS in diestrus/metestrus or control vs ELS in proestrus/estrus in the OAL model. Two-way RM ANOVAs were used to assess the effects of estrus cycling on visceral sensitivity in all groups followed by a Bonferroni post hoc test (Graphpad Prism, 6.0c, La Jolla, CA).

## Results

### Weaning weights

**Fig. 1:**
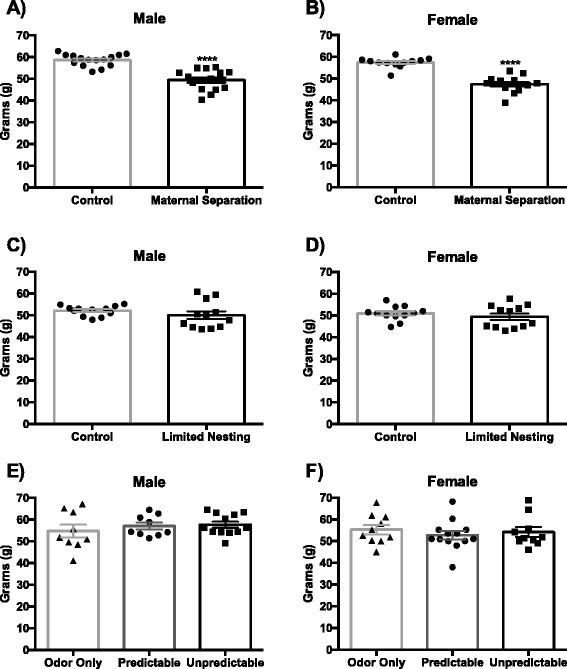
Weaning weights following ELS exposure. Maternal separation causes a reduction in weaning weights in both male and female (*n* = 12–15) animals, ****p < 0.0001 (**a**, **b**). Limited nesting (*n* = 12) and odor attachment learning (*n* = 9–13) do not alter neonate-weaning weights (**c**–**f**)

All pups that underwent each form of ELS were weaned and weighed on PND 22 to determine the effect of neonatal experience on body weight. Removal of pups from the dam during the MS protocol of ELS had a significant effect on growth prior to weaning. Following 180 min of MS per day from PND2-14, both male (*p* < 0.0001) and female (*p* < 0.0001) pups had lower weaning weights compared to their non-separated counterparts (Fig. 1a, b). In contrast, pups that experienced LN or OAL did not exhibit differences in body weight at weaning compared to control animals (Fig. 1c–f). There was no effect of sex in any of the ELS paradigms on weaning weights.

### Somatic sensitivity

**Fig. 2:**
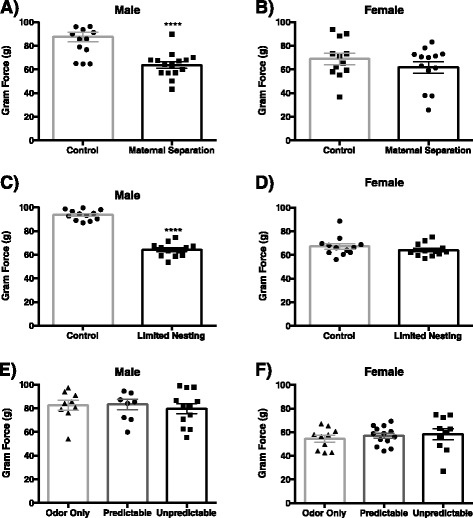
Somatic sensitivity to von Frey filaments in adulthood following ELS. Maternal separation (*n* = 12–15) (**a**, **b**) and limited nesting (*n* = 12) (**c**, **d**) induce male-specific increases in somatic sensitivity in adulthood, ****p < 0.0001. Odor attachment learning (*n* = 9–13) (**e**, **f**) does not alter the force necessary for withdrawal in any group

Following ELS, adult male and female rats from all treatment groups were evaluated for somatic sensitivity by quantifying the force necessary to elicit a hindpaw withdrawal in response to a von Frey filament probe. Our experiments revealed a main effect of sex (*F*_(1,51)_ = 6.35, *p* < 0.0001) and stress (*F*_(1,51)_ = 4.18, *p* < 0.05) with an interaction between sex and stress (*F*_(1,51)_ = 14.34, *p* < 0.05) in the MS model. Post hoc analysis revealed that adult males previously exposed to MS showed a decreased threshold for hindpaw withdrawal compared to controls (*p* < 0.0001) (Fig. 2a), whereas females exposed to MS exhibited no somatic allodynia as adults (*p* > 0.05) (Fig. 2b). Following neonatal LN, there was a significant effect of sex (*F*_(1,44)_ = 55.48, *p* < 0.0001) and stress (*F*_(1,44)_ = 84.3, *p* < 0.0001) as well as an interaction between sex and stress (*F*_(1,44)_ = 55.97, *p* < 0.0001) on somatic sensitivity. Similar to the MS model, post hoc analysis revealed that the LN model of ELS induced male specific somatic hypersensitivity, with a decreased threshold for withdrawal compared to normal nested controls (*p* < 0.0001) (Fig. 2c) while adult females from the LN group exhibited responses to von Frey probing similar to their control counterparts (*p* > 0.05) (Fig. 2d). In contrast, although there was a main effect of sex (*F*_(2,57)_ = 63.89, *p* < 0.0001), the OAL model of ELS did not induce changes in behavioral responses to somatic stimuli in male (*p* > 0.05) or female (*p* > 0.05) animals across treatments: predictable, unpredictable, or odor-only controls (Fig. 2e, f). Although comparison of metestrus/diestrus vs proestrus/estrus within each group revealed significant changes in withdrawal threshold due to cycle, comparison of control vs ELS in diestrus/metestrus or control vs ELS in proestrus/estrus revealed no differences in somatic sensitivity between control and ELS-treated animals (Table 2). Therefore, our results were unchanged by the analysis of somatic sensitivity in either the diestrus/metestrus or proestrus/estrus phases of the estrous cycle.

**Table 2:** Somatic sensitivity in adult female animals following ELS across the estrus cycle

Phase	Treatment	Gram force (g) to elicit withdrawal
Metestrus/diestrus	Control	56.2 ± 4.2
	Maternal separation	50.7 ± 6.3
	Control	60.2 ± 1.4
	Limited nesting	59.2 ± 0.8
	Odor only	51.5 ± 2.7
	Predictable	53.2 ± 2.0
	Unpredictable	49.8 ± 5.3
Proestrus/estrus	Control	81.8 ± 4.1***
	Maternal separation	74.2 ± 1.8***
	Control	70.7 ± 2.8**
	Limited nesting	66.7 ± 1.7**
	Odor only	66.3 ± 0.9**
	Predictable	65.8 ± 1.4***
	Unpredictable	71.3 ± 2.8**

Mean ± SEM

**p < 0.05 vs same cycle control

***p < 0.05 metestrus/diestrus vs proestrus/estrus within same group

### Visceral sensitivity

**Fig. 3:**
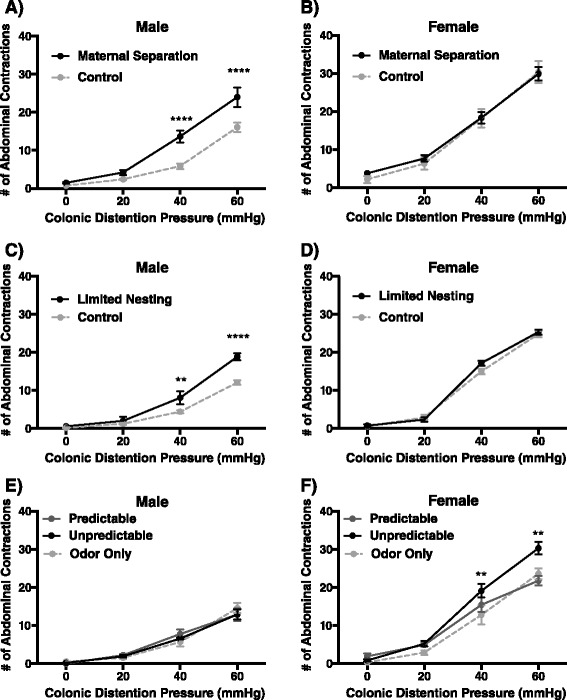
ELS exposure leads to sexually dimorphic development of visceral hypersensitivity in adulthood. Maternal separation (*n* = 12–15) (**a**, **b**) and limited nesting (*n* = 12) (**c**, **d**) induce male-specific increases in the visceromotor response to colorectal distension in adult rats, **p < 0.01, ****p < 0.0001. Odor attachment learning (*n* = 9–13) (**e**, **f**) leads to female-specific visceral hypersensitivity in the unpredictable group, **p < 0.01

Male and female animals previously exposed to MS, LN, or OAL in early life were assessed for abnormalities in visceral nociception by quantifying the number of abdominal contractions in response to isobaric colonic distension at graded pressures of 0, 20, 40, and 60 mmHg. Following neonatal MS, statistical analysis revealed a significant effect of sex (*F*_(1,53)_ = 28.46, *p* < 0.0001), a significant effect of pressure (*F*_(3,159)_ = 307.9, *p* < 0.0001), and an interaction between sex and pressure (*F*_(3,159)_ = 10.85, *p* < 0.0001). We found that ELS in the form of MS induced visceral hypersensitivity as adult males compared to non-separated controls revealing a significant main effects of pressure (*F*_(3,87)_ = 144.40, *p* < 0.0001) and stress (*F*_(1,29)_ = 17.10, *p* < 0.001) with a significant interaction between pressure and stress (*F*_(3,87)_ = 7.70, *p* < 0.001). Bonferonni post hoc testing revealed a significant difference in visceromotor response to CRD at 40 mmHg (*p* < 0.0001) and 60 mmHg (*p* < 0.0001) in MS-treated males compared to non-separated controls (Fig. 3a). In females exposed to neonatal MS, there was no significant effect of stress on visceral sensitivity compared to controls although there was a significant effect of pressure (*F*_(3,69)_ = 192.4, *p* < 0.0001) (Fig. 3b). Following neonatal LN, statistical analysis revealed a significant effect of sex (*F*_(1,46)_ = 74.72, *p* < 0.0001), a significant effect of pressure (*F*_(3,138)_ = 742.7, *p* < 0.0001), and an interaction between sex and pressure (*F*_(3,138)_ = 61.49, *p* < 0.0001). Similar to MS, following LN treatment adult male animals exhibited visceral hypersensitivity at 40 mmHg (*p* < 0.01) and 60 mmHg (*p* < 0.0001) compared to control males as demonstrated by significant main effect of pressure (*F*_(3,66)_ = 255.20, *p* < 0.0001) and stress (*F*_(1,22)_ = 10.04, *p* < 0.01) and a significant interaction between pressure and treatment (*F*_(3,66)_ = 11.91, *p* < 0.0001), whereas CRD in females revealed a significant main effect of pressure (*F*_(3,66)_ = 780.40, *p* < 0.0001) but no effect of stress on visceral sensitivity (Fig. 3c, d). In contrast to the MS and LN models, the OAL model exhibits both a context and sex-dependent increase in the development of visceral hypersensitivity, where only adult female animals experiencing unpredictable ELS exhibit an exaggerated VMR to CRD at 40 (*p* < 0.01) and 60 mmHg (*p* < 0.01) compared to odor-only controls with a significant main effect of pressure (*F*_(3,90)_ = 315.0, *p* < 0.0001) and stress (*F*_(2,30)_ = 4.868, *p* < 0.05) and a significant interaction between pressure and stress (*F*_(6,90)_ = 4.60, *p* < 0.001). Statistical analysis also revealed a significant effect of sex (*F*_(1,61)_ = 76.68, *p* < 0.0001), a significant effect of pressure (*F*_(3,183)_ = 405.1, *p* < 0.0001), and an interaction between sex and pressure (*F*_(3,183)_ = 37.76, *p* < 0.0001). Adult males and females who experienced predictable ELS exhibit responses to CRD that parallel their respective odor-only control counterparts (Fig. 3e, f). Comparison of metestrus/diestrus vs proestrus/estrus within each group revealed a significant effect of cycle within the limited nesting control group (*F*_(1,44)_ = 11.5, *p* < 0.01) and the predictable ELS group (*F*_(1,11)_ = 27.57, *p* < 0.001), as well as significant post hoc differences at 40 and 60 mmHg (Table 3). However, comparison of control vs ELS in diestrus/metestrus or control vs ELS in proestrus/estrus revealed no differences in visceral sensitivity between control and ELS animals in the MS or LN models while the heightened sensitivity of females exposed to unpredictable ELS was maintained (Table 3). Together, our results demonstrate that visceral pain is directly dependent on the context of the ELS and is linked with sex differences in the development of abnormalities in pain perception.

**Table 3:** Visceral sensitivity in adult female animals following ELS across the estrus cycle

Phase	Treatment	0 mmHg	20 mmHg	40 mmHg	60 mmHg
Metestrus/diestrus	Control	1.1 ± 0.3	5.1 ± 1.6	16.4 ± 3.1	27.3 ± 3.3
	Maternal separation	4.3 ± 1.4	8.3 ± 1.6	17.0 ± 2.4	28.8 ± 1.2
	Control	0.6 ± 0.2	2.9 ± 0.8	13.3 ± 0.7	23.6 ± 1.9
	Limited nesting	0.5 ± 0.3	3.5 ± 1.3	15.8 ± 0.9	23.3 ± 1.4
	Odor only	0.2 ± 0.2	2.5 ± 0.8	12.2 ± 1.5	21.2 ± 1.5
	Predictable	1.1 ± 0.3	4.7 ± 0.6	12.1 ± 1.3	19.4 ± 0.9
	Unpredictable	0.5 ± 0.3	5.3 ± 1.3	14.8 ± 3.3*	27.0 ± 3.7*
Proestrus/estrus	Control	3.6 ± 2.1	8.0 ± 3.0	20.8 ± 4.1	34.6 ± 4.9
	Maternal separation	3.3 ± 0.9	7.1 ± 0.9	19.6 ± 2.1	30.9 ± 3.1
	Control	0.6 ± 0.6	3.0 ± 1.3	17.4 ± 0.6***	26.6 ± 1.3**
	Limited nesting	0.9 ± 0.5	1.8 ± 0.4	18.9 ± 0.9	26.4 ± 2.4
	Odor only	0.5 ± 0.3	3.5 ± 1.0	13.8 ± 6.3	27.3 ± 1.3
	Predictable	3.8 ± 2.2	5.5 ± 0.3	23.0 ± 3.3***	27.0 ± 1.7***
	Unpredictable	1.0 ± 0.4	5.2 ± 1.0	22.0 ± 0.9	32.5 ± 2.6*

Mean ± SEM

*p < 0.05 vs same cycle control, **p < 0.05 metestrus/diestrus vs proestrus/estrus within same group

## Discussion

Previous studies have shown that a history of ELS is associated with chronic pain in adulthood, most commonly seen in females [6]. In the current study, we aimed to test the hypothesis that the development of chronic pain in adulthood is influenced by sex differences in either vulnerability or resilience to a specific ELS experience. To address this hypothesis, we employed three validated rodent models of ELS that attempt to mimic different forms of adverse early life experience. The first rodent model of maternal separation was used to emulate a situation of neglect and abuse via separation of pups from the dam 3 h/day PND 2–14 [14]. A second model of ELS, limited nesting, was employed to mimic maltreatment in an impoverished environment by removing the bedding material and limiting the resources available for the dam to build a nest [25]. While both MS- and LN-induced maternal stress create an early life adverse experience indirectly by inducing alterations in nurturing behavior of the dam, a third model of ELS, odor attachment learning, utilizes the manipulation of the pups directly. OAL recapitulates attachment to an abusive caregiver through patterning of a classical conditioning protocol wherein pups learn to prefer the presented odor or “abusive caregiver” [19]. While the patterned or paired odor/shock presentation is predictable, a second unpatterned odor/shock presentation allows for an unpredictable ELS that does not induce caregiver attachment [19]. A novel aspect to our study was that in each of the three models, we focused on the importance of sex-related differences in the development of enhanced pain behaviors in adulthood following ELS. Our study provides experimental evidence that males and females exhibit marked differences in pain behaviors in adulthood that are dependent on the specific neonatal ELS experience.

Sex differences in pain thresholds between male and female animals have been well characterized, including variations in female pain responses throughout the estrus cycle [26]. Cyclical fluctuations in female animal pain responses parallel patient studies indicating that women experience worsening of symptoms during menses that correlate to increases in female hormones [27–29]. In support, the current series of experiments demonstrated variation in pain responses of female animals across the estrus cycle where females in the proestrus/estrus were more sensitive to painful stimuli than those in the metestrus/diestrus phases. Clinical studies suggest that females disproportionately experience functional pain disorders compared to their male counterparts [30, 31] and evidence suggests that symptoms may lessen following menopause [32, 33]. While previous research sheds light on the differences in male and female pain thresholds, there is little data to explain why females disproportionately develop functional pain disorders. Estrogen and progesterone have been indicated as modulators of decreased pain thresholds in female animals compared to their male counterparts, but the exact mechanism by which female hormones may increase the risk of developing chronic pain remains unknown [34, 35]. Thus, in the current study, we focused on differences in pain thresholds between male and female animals as well as whether ELS induces sex-specific alterations in pain perception that could improve our understanding of the female predominance in functional pain disorders.

Patients who experience functional pain disorders such as IBS experience a myriad of symptoms including somatic and/or visceral hyperalgesia. Our data identifies ELS in rodents as a relevant tool for the study of both somatic and visceral hypersensitivity in adulthood that develops as a result of early adverse experience. To our knowledge, there are no previous studies investigating the effect of MS on mechanical somatic sensitivity, and therefore, this series of experiments provides a greater understanding of pain-related consequences in adult animals. Previously, somatic allodynia following ELS was investigated in the LN model to fully explore sex-related differences in somatic withdrawal responses in adulthood using Sprague Dawley rats [17]. In the current study, following MS and LN exposure, adult male rats exhibited somatic allodynia, while female rats exposed to the same ELS paradigms displayed similar responses to non-separated or normal nested controls. Having shown that MS and LN induced male-specific alterations in somatic sensitivity, we expanded our observations by investigating visceral sensitivity within these models. The current data set confirmed the development of visceral hypersensitivity in male animals following MS exposure [36] but also identified a previously unexplored sex difference in Long Evans rats, wherein female animals exhibit pain thresholds for visceral or somatic assessments consistent with controls. The effects of MS on pain responses in adulthood complement our previous work showing that LN exposure induces male-specific changes in adult visceral sensitivity, while female animals were seemingly unaffected [17]. The male-specific effect of LN shown previously was mirrored in the current study using the Long Evans strain of rat. In the OAL model of ELS, the assessment of somatic sensitivity revealed no differences in mechanosensitivity following ELS in male or female animals compared to odor-only controls. As confirmed in the current study, our laboratory has previously shown a female-specific increase in visceral pain perception that is context dependent following OAL exposure [18]. Taken together, our study supports sex-specific differences in vulnerability to ELS based on the specific type of adverse experience. Furthermore, our data also implies that there may be differential mechanisms underlying the development of somatic and visceral pain despite shared nociceptive circuitry. In support, clinical studies have shown exaggerated bilateral activation of the anterior cingulate cortex in response to somatic stimuli while visceral pain evoked increased activation of bilateral inferior primary somatosensory cortex, primary motor cortex, and an anterior locus within the anterior cingulate cortex [37]. In rodents, the periaqueductal gray has also been indicated as nuclei for the divergence of nociceptive information with the lateral PAG modulating somatic sensitivity, while the ventrolateral PAG was activated in response to visceral stimuli [38, 39]. Thus, brain and brainstem modulation of nociceptive information may play a role in the divergence of somatic and visceral pain perception.

ELS has the potential to disrupt key neurodevelopmental time points during normal neonate development. One such time point is the stress hyporesponsive period, which is characterized by diminished ability to experience stress or fear in response to aversive stimuli. The hyporesponsive period is ended around PND 14 by the release of corticosterone, which activates the amygdala and initiates the ability of the brain to facilitate stress and fear [40]. Early activation of neurocircuitry via corticosterone release due to ELS exposure represents a change in normal development that could alter activity within brain regions responsible for modulating pain perception such as the amygdala [23, 41–43]. Previous studies indicate that MS and LN exposure lead to early release of corticosterone in neonates prior to the end of the stress hyporesponsive period [25, 44]. In contrast, the OAL model does not elicit an early release of corticosterone nor does it prematurely end the hyporesponsive period [45]. Thus, differences in the long-term consequences of these ELS paradigms could be a result of their respective influence on amygdala activation. Although the timing of ELS differs slightly among paradigms, it is unlikely that timing alone underlies abnormalities in pain perception. A second potential mechanism for alterations in pain-related circuitry involves sex-specific modulation of pain via hormone release. Male and female sex hormone receptors have been identified within nuclei that modulate stress and pain, thus providing a platform for gonadal hormones to influence behavior in a sexually dimorphic manner [46–48]. Sexually dimorphic responses to psychological or physical insults have been characterized in adult animals including more robust and long lasting corticosterone responses to physical stress in females compared to their male counterparts [48, 49]. Following ELS, adult animals have also been shown to exhibit exaggerated stress responses indicative of altered stress axis function as a result of early adverse experience [44]. Together, these studies indicate that disruption of normal development caused by ELS may lead to divergent stress responses that are compounded by the effects of gonadal hormones in males and females, ultimately allowing for the sexually dimorphic development of chronic pain in adulthood. While stress responses were not investigated in this series of experiments, it remains an interesting future study to examine corticosterone responses to stress in adulthood following each ELS paradigm.

A key indicator of healthy growth and nutrition in neonates is the weight of the pup at weaning. In the MS model, following removal of pups from the dam during PND2-14, we confirmed previous observations that MS causes a decrease in the weaning weights of separated neonates [44]. In contrast, we showed that LN or OAL exposure does not alter pup weight at weaning compared to controls. These findings suggest that malnutrition could be a confounding factor for the interpretation of long-term changes in behavior following MS treatment. A lack of nutrition during critical points in development could alter different circuitry than ELS treatment alone [50]. While decreased weaning weight certainly serves as a point of consideration, malnutrition is commonly present in the human population concurrent with situations of adversity. Importantly, although there is some variation between weaning weights of control animals, we consider this to be natural variability between cohorts as neonates from dams designated for the control and treatment group for each paradigm were cross-fostered on PND 1. Differences in time of year in which rodents are born or litter size have also been suggested as potential factors for variability in weaning weights. However, overlap between experimental groups in the current study and consistency in weaning weights between litters of difference sizes, as evidenced by our data set, suggest these are not confounding factors in our interpretation. Therefore, comparison of experimental data following MS versus LN or OAL may reveal alternative origins for the development of long-term pathologies and be a useful tool in translating rodent data into the human population.

A significant concern in the comparison and interpretation of previous studies is that ELS has been investigated in a myriad of experimental models utilizing different rodent strains. The MS and OAL models were originally developed in Long Evans rats [45, 51], while the LN model was developed in the Sprague Dawley strain of rats [25]. In an effort to use the original rodent strain for the majority of models assessed in the current set of experiments, we chose Long Evans rats for this study. Additionally, evidence suggests that Long Evans rats learn more efficiently than other strains such as Sprague Dawley or Wistar rats, making the use of Long Evans rats important for our classical conditioning ELS paradigm [52]. In the MS model, the use of alternative rodent strains such as Wistar rats, indicated visceral hypersensitivity in both male and female rats [53] which were in part consistent with subsequent studies of adult male Long Evans rats who exhibited visceral hypersensitivity [36]. In the LN model, our previous publication using Sprague Dawley rats indicated that the development of viscerosomatic hypersensitivity was specific to adult male rodents while females were unaffected [17]. However, a similar study in Wistar rats indicated that LN elevated visceral pain responsiveness in both male and female adult rodents at noxious levels distension [54]. To date, OAL has only been investigated in the Long Evans rodent strain resulting in female-specific visceral hypersensitivity observed following an unpredictable ELS [18]. When interpreting the large body of research regarding long-term effects of ELS, differences in strain are an important consideration because a simple change in genetic profile may convey vulnerability or resilience to a specific ELS exposure or introduce the potential for compensatory mechanisms that do not exist in other rodents. Therefore, genetic differences among rodent strains could explain variability in the long-term consequences of ELS across studies. Alternatively, conflicting or variable results may mirror the diversity of the human population and ultimately allow researchers to define common target downstream of divergent mechanisms. Whether variability among studies occurs because of paradigm, sex, or strain, the identification of common downstream targets could be beneficial in the development of new therapeutic approaches that may apply to a broader patient base independent of ELS experience, sex, or genetic background.

## Conclusions

In summary, the current study implicates both the type of ELS exposure and sex as pivotal factors in modulating the development of enhanced pain behaviors in adulthood. Of importance to the interpretation of our findings, we have eliminated the confounding influences of rat strain by performing all our experiments in Long Evans rats and advanced the literature by including both male and female animals in our analysis of all three ELS models. While our study and others indicate ELS as a risk factor for the development of long-term alterations in pain, the current investigation strongly suggests that variations in paradigm have the potential to induce chronic pain through differing mechanisms, which are sex-dependent. Given the well-established female predominance among patients who suffer from functional pain disorders, our study suggests that the female-specific induction of visceral hypersensitivity following OAL may most closely mimic the clinical situation. However, each model of ELS utilized in our study represents a unique facet of the complex human experience of ELS and provides an avenue for the development of future therapeutic approaches tailored to the needs of patients experiencing chronic pain.

## Abbreviations

ANOVA, analysis of variance; CRD, colorectal distension; ELS, early life stress; IBS, irritable bowel syndrome; LN, limited nesting; MS, maternal separation; OAL, odor attachment learning; PND, postnatal day; VMR, visceromotor response
